# Isolation, Culture, and Functional Characterization of Human Embryonic Stem Cells: Current Trends and Challenges

**DOI:** 10.1155/2018/1429351

**Published:** 2018-08-26

**Authors:** Firdos Alam Khan, Dana Almohazey, Munthar Alomari, Sarah Ameen Almofty

**Affiliations:** Department of Stem Cell Biology, Institute for Research and Medical Consultations, Imam Abdulrahman Bin Faisal University, Post Box No. 1982, Dammam 31441, Saudi Arabia

## Abstract

Human embryonic stem cells (hESCs) hold great potential for the treatment of various degenerative diseases. Pluripotent hESCs have a great ability to undergo unlimited self-renewal in culture and to differentiate into all cell types in the body. The journey of hESC research is not that smooth, as it has faced several challenges which are limited to not only tumor formation and immunorejection but also social, ethical, and political aspects. The isolation of hESCs from the human embryo is considered highly objectionable as it requires the destruction of the human embryo. The issue was debated and discussed in both public and government platforms, which led to banning of hESC research in many countries around the world. The banning has negatively affected the progress of hESC research as many federal governments around the world stopped research funding. Afterward, some countries lifted the ban and allowed the funding in hESC research, but the damage has already been done on the progress of research. Under these unfavorable conditions, still some progress was made to isolate, culture, and characterize hESCs using different strategies. In this review, we have summarized various strategies used to successfully isolate, culture, and characterize hESCs. Finally, hESCs hold a great promise for clinical applications with proper strategies to minimize the teratoma formation and immunorejection and better cell transplantation strategies.

## 1. Embryonic Stem Cells: Early Discovery and Isolation Procedure

Embryonic stem cells (ESCs) were first isolated from mouse embryos in 1981, and the word “embryonic stem cell” was first coined by Gail R. Martin. Nonetheless, the world came to know about ESCs with the breakthrough discovery in 1998, where Thomson and his team showed for the first time a technique to isolate hESCs from human embryos. Thereafter, researchers have demonstrated that hESCs have an ability to differentiate into all body cells, including beta cells of the islets of Langerhans [1], neural cells [2], cardiomyocytes [3], and hepatocyte-like cells [4]. The pluripotent capabilities of hESCs have given hope to millions of patients who are suffering from diabetes, Parkinson's disease, cardiovascular disease, and liver diseases. Considering hESCs having great therapeutic potentials, several hESC lines were generated across the world. One of the challenges of the hESCs was the method of isolation of stem cells from the human embryo, as hESCs can only be obtained from the inner cell mass (ICM) of human embryos [5]. Researchers reported that ICM can be obtained from either fresh or frozen human embryos [5–7]. Thereafter, several methods were developed to isolate ICM from a single human embryo, which include mechanical dissection, where ICM is isolated by mechanical pressure [6, 7]. The ICM can also be isolated by using laser dissection [8, 9] and by using immunosurgery procedures [10–12]. There are various benefits of using an immunosurgery procedure to isolate ICM, but this also carries some disadvantages. For example, the immunosurgery procedure requires the culture media which contain guinea pig serum; hence, the use of animal serum makes the immunosurgery technique not suitable for the generation of clinical-grade hESC lines [13]. In another method, hESC lines can be isolated from ICM by microdissection of human blastocysts using fine needles. Laser-assisted biopsy is also the most promising technique for xeno-free isolation of the ICM [9, 14]. After ICM isolation, the stems cells are grown to generate the ESCs using feeder layers, extracellular matrices, proteins, peptides, and synthetic polymers [9, 14]. Advantages and disadvantages of various methods of ICM isolation are summarized in Table 1.

The isolation of ICM requires destruction of human embryos which has raised serious ethical concerns [15]. To satisfy the ethical issue, researchers demonstrated an alternate approach to isolate hESCs from a single blastomere without killing or destroying the human embryo. For example, during preimplantation genetic testing, embryo biopsy carrying a single blastomere can be obtained from patients ([16, 17]; Klimanskaya et al., 2009). It has been reported that 5 hESC lines were successfully obtained from a single blastomere biopsy [16]. The success of obtaining good-quality hESCs depends on the quality of blastocysts and isolation procedures and culture conditions. It was reported that 2 hESC lines were obtained from 4 blastocysts, whereas only 3 hESC lines could be isolated from 13 blastocysts and, in some cases, only 3 hESC lines could be isolated from 58 blastocysts [13]. These differences in isolation of hESC lines from different blastocysts are mainly due to the quality of embryos and also depend on the method of embryo isolation and culture protocols [18, 19]. For example, if an embryo is obtained through an *in vitro* fertilization method, then there is a great possibility that embryos will have a high incidence of postzygotic chromosomal abnormalities which may eventually give poor quality of hESCs [13].

In mice, pluripotent stem cells can also be derived from the epiblast of post-implantation-stage embryos, commonly known as epiblast stem cells. These pluripotent stem cells show primed characteristics and are highly dependent upon the activation of FGF and activin signalling pathways for their self-renewal [20, 21]. Consequently, three distinct pluripotent conditions, namely, naive, primed, and ground pluripotency conditions, have been defined in mice [22].

## 2. Culturing of hESCs with or without Feeder Cells

Once the blastomere is collected, it is normally cocultured with the parental biopsy embryo in the medium containing fibronectin and laminin. The addition of laminin in the culture media is important for the formation of embryonic stem cell- (ESC-) like aggregates. In addition, there are reports which suggest that addition of serum-free media and fibroblast growth factors enhance stem cell proliferation and prevent embryonic stem cells from undergoing differentiation [23, 24]. We have briefly described various culture conditions which have been used to improve both quality and quantity of generation of hESCs.

### 2.1. Mouse Feeder Cells to Grow hESCs

**Figure 1:**
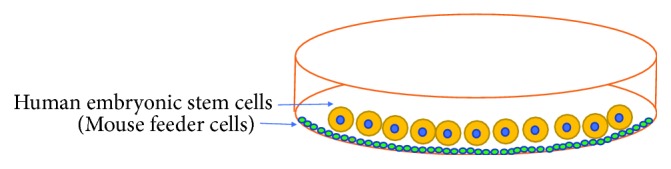
Culture of human embryonic stem cells: human embryonic stem cells can be cultured on the mouse feeder cells (MEF).

Mouse embryonic fibroblast (MEF) cells or mouse feeder cells are considered most important elements for hESCs because MEF provides favorable condition for growth and expansion of hESCs (Figure 1). It has been reported that MEFs are very important for the successful generation of hESC lines [11, 12]. In addition, all early hESC lines were grown in the media containing growth factors and cytokines secreted by MEF cells, and these growth factors and cytokines are necessary to maintain the pluripotency of the stem cells. As MEF was derived from a mouse source, it has posed serious ethical or health issues for hESCs. Moreover, the use of animal-based cells can transmit animal-derived infectious pathogens to hESCs and make them not suitable for human utilization. It has been reported that MEF cells contain viral particles that are capable of infecting hESCs during culture [25]. Furthermore, some researchers have used bovine serum to culture hESCs, but the use of animal-derived serum can transmit prion and animal viruses in embryonic stem cell culture [26]. It has been reported that the animal-based cells and serums can transmit viruses and other pathogens into embryonic stem cells through cell-cell interaction during *in vitro* culture [27, 28]. Furthermore, these pathogenic molecules can contaminate the entire hESC culture. In case hESCs are contaminated with such pathogens, the contamination issue may persist even hESCs are later transferred to nonanimal free culture condition. Another problem with mouse feeder cells and animal-derived serum/proteins is that they also contain nonhuman sialic acid (Neu5GC) which can also pose a serious contamination problem to hESCs [29]. For example, it was reported that animal-derived sialic acid metabolically entered the cell surface of hESCs and contaminated embryonic stem cells [29].

### 2.2. Nonanimal Feeder Cells to Grow hESCs

To avoid animal-based products and cross-species contaminations, researchers have developed culture media which do not contain animal components and at the same time supported the growth and expansion of embryonic stem cells. It has been reported that human cells can be used for hESC culture; for example, human fallopian tube cells [30], fetal foreskin [31], fetal muscle and skin [32], transgenic fetal liver stromal cells [33], bone marrow [34], umbilical cord [35], placental cells [36, 37], and endometrial cells [36, 38, 39] have been reported to support stem cell culture and expansion. Among these human cells, human umbilical stromal cells offer a better source of feeder cells that can also be collected using a noninvasive method, whereas the usage of foreskin-, fetal-, or bone marrow-derived feeder layers raises some ethical concerns.

Besides feeder cells, human cell lines also provide an alternative to mouse feeder cells. Recently, several hESC lines were derived and propagated using a commercially available human foreskin fibroblast line [40, 41]. Endometrial cells also proved to be effective for *in vitro* culture of stem cells [38, 39, 42]. Another way to eliminate the risk of animal pathogen contamination is the use of feeder layers derived from the human stem cell line [43, 44]. Basic fibroblast growth factor (bFGF) is shown to be endogenously produced by human feeder cells used in hESC culture ([33, 45]; Liu et al., 2014). These feeder cells also secrete TGF*β* and activin A which are involved in maintaining the pluripotency of ICM [46, 47]. Despite having various benefits, feeder cell-dependent hESC culture has many limitations; for example, maintenance of feeder layers is laborious with too much variation between feeder cell populations. This disparity can negatively affect the hESC claim for human application.

### 2.3. Feeder-Free Culture to Grow hESCs

**Figure 2:**
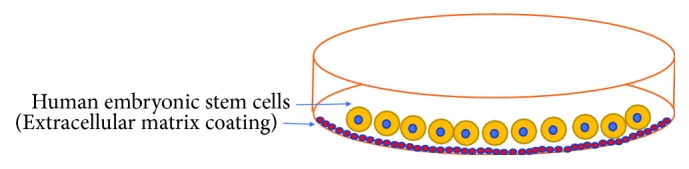
Culture of human embryonic stem cells: human embryonic stem cells can be cultured on the extracellular matrix such as Matrigel.

As both animal and human feeder cells have limitations, researchers have explored and have successfully designed chemically defined culture media to culture hESCs, and the best thing about the defined media is that they do not contain any feeder cells. One of the first approaches tried for feeder-free growth media was the use of extracellular matrix proteins along with growth factors to create an *in vitro* culture condition for the stem cell proliferation and renewal (Figure 2). Among these proteins, Matrigel [48] was mostly used in combination with growth factors or conditioned medium to culture hESCs [48, 49]. Despite various benefits, Matrigel found to have too many variations in its compositions which posed problems to hESC culture. The use of Matrigel also raises clinical issues as few batches of Matrigel have been reported to be contaminated with the single-stranded mouse RNA virus-lactate dehydrogenase elevating virus [50]. Besides Matrigel, fibronectin, laminin, and collagen type IV have also been good candidates for xeno-free hESC culture, and cells could grow up to 20 passages [51, 52]. Reference [53] reported that human placenta-derived ICM was used to culture hESCs, and they found strong genetic stability for 40 passages. Moreover, hESCs were also grown in xeno-free culture media up to 80 passages [40, 41].

Undoubtedly, the use of chemically defined media along with proteins has significantly improved the culture of hESCs. In addition, different proteins and recombinant proteins were also used to enhance hESC culture under xeno-free condition. Among those were E-cadherin, E-cadherin/laminin 521, and kinase inhibitors along with bFGF which are known to cause robust proliferation of stem cells under xeno-free conditions [54]. Synthetically designed bed surface was also used to stimulate stem cell culture (Melkoumian et al., 2010); for example, Corning Synthemax Surface, a synthetic acrylate surface conjugated with vitronectin, was shown to enhance not only hESC colonies but also expansion of stem cells (Kawase et al., 2014). Wu et al. recently described the use of novel synthetic material isolated from spider silk proteins as a suitable substrate to stimulate hESC culture (Wu et al., 2014). Numerous polymer-based synthetic surfaces have been also reported to support the growth and expansion of hESC lines (Melkoumian et al., 2010; Brafman et al., 2010; Villa-Diazet al., 2013). The list of different chemicals used to enhance culture of hESCs is shown in Table 2.

## 3. Multilineage Potential of hESCs

**Figure 3:**
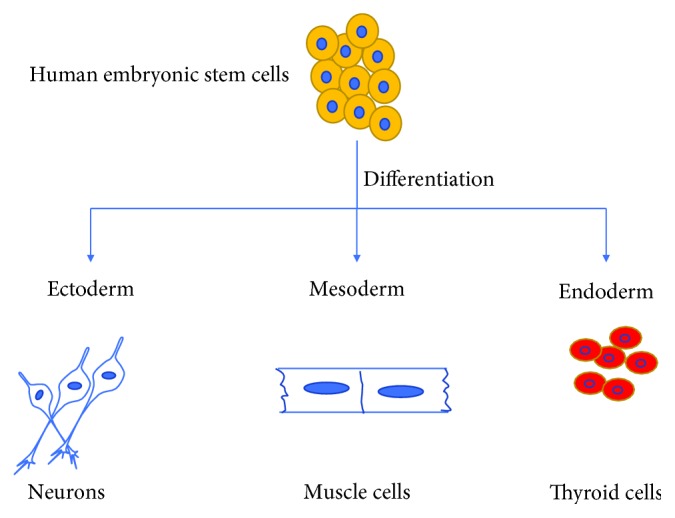
Multilineage potential of human embryonic stem cells: human embryonic stem cells can be differentiated into three germ-layers such as ectoderm, mesoderm, and endoderm.

One of the utmost characteristics of hESCs is to differentiate into all three lineages such ectoderm, mesoderm, and endoderm (Figure 3). As hESCs are pluripotent stem cells, they have unique capabilities to differentiate into all kinds of body cells; for example, hESCs can be differentiated into neurons, cardiac cells, hepatocytes, and muscle cells. It has been reported that hESCs first form embryoid bodies which are basically structured with three germ layers. These embryoid bodies are formed by pluripotent hESCs grown in 3-dimensional (3D) culture and expressed genetic markers for all three germ layers [55–57]. Pluripotent hESCs have a tremendous ability to differentiate (Table 3) into adrenal cells and keratinocytes [58], insulin-producing cells [59], neuronal cells [60, 61], cardiac cells [62], liver cells [63], and islet-like organoid [64]. Certain growth factors such as retinoic acid and nerve growth factors are being used to induce hESCs to differentiate into functional neurons. Moreover, some lineage-specific growth factors are being used for the differentiation into cardiomyocytes, hepatocytes, skeletal muscles, pancreatic cells, and kidney cells. These differentiated cells are also being tested for examining their functionality in both *in vitro* and *in vivo* conditions. This multilineage potential of hESCs has proved to be vital for cell-based therapy to treat different degenerative diseases. While it is easy to differentiate different types of cells from hESCs, it is difficult to get a large number of differentiated mature cells for therapeutic applications [65]. To obtain large, mature, and functional differentiated cells, the culture media should contain lineage-specific growth factors. It is also important to generate large quantities of cells from hESCs as they are required for cell transplantation, and this can be achieved by culturing the hESCs and differentiated cells in a bioreactor under control condition [66].

## 4. Testing of hESCs Using *In Vitro* and *In Vivo* Models

After a successful differentiation of hESCs into various cell types, the next logical step is to examine whether derived differentiated cells have some functionality or not. The functionality of stem cells and differentiated precursor or mature cells was examined extensively in both *in vitro* and *in vivo* conditions. The functionality of differentiated neurons, cardiomyocytes, hepatocytes, and other types of cells was tested in various animal models [67]. It was found that transplantation of neurons in the animal model of Parkinson's disease caused a partial recovery of the function [68, 69]. The transplantation of hESCs and their differentiated cells was tested in the animal models of cardiovascular disease, stroke, diabetes, and spinal cord injury [67]. Among animals, small rodents such as rats and mice have been a species of choice to study cell transplantation. Moreover, small rodents are effortlessly accessible and can be easily manipulated both surgically and genetically. Despite various benefits of small rodents, the ability of mouse/rat experiments to predict the efficacy of stem cell-based therapy remains contentious [70] as many mouse/rat models do not represent human disease phenotypes. To overcome this issue, researchers have started working on the large animals which are close to human anatomy and physiology. Among large animals, dogs, goats, sheep, and nonhuman primates are considered better models than mice/rats for the stem cell testing [70]. One of the main advantages of using large animals is their longer life span, and many anatomical, physiological parameters are much closer to humans [70]. Though these animal models demonstrate the effective delivery of stem cells in the host tissues, the complete functional and behavioral recovery is still not achieved. Further research is required to develop animal models which are close to human disease.

Despite this progress in hESC research, one important challenge of hESC-based cell therapy is the allogeneic immune rejection of hESC-derived cells by recipients [71]. It was found that within a week, all the transplanted stem cells died due to the strong host immune response generated in animals. To stop the death of the transplanted stem cells, animals were injected with immunosuppressors to suppress the immunity triggered by stem cell transplantation. Surprisingly, when the animals were given immunosuppressors or drugs like tacrolimus and sirolimus, the hESCs could survive only for 28 days and started dying thereafter [72]. While we do not know the reason for this, a lack of understanding of cell-cell interaction could be one of the reasons. It is important to test hESCs or differentiated cells under *in vitro* condition prior to animal testing. *In vitro* models provide better opportunities to study the cell-cell interaction, cell migration, or cell integration with a great detailed manner, which perhaps is very difficult to study in the animals. This problem could be mitigated by a recent breakthrough in the technology of induced pluripotent stem cells (iPSCs) by nuclear reprogramming of patient-specific somatic cells with defined factors, which could become a renewable source of autologous cells for cell therapy. One key advantage of iPSCs for human cell therapy is that patient-specific iPSCs are autologous, and, therefore, it has been assumed that the cells derived from them can be transplanted into the same patient without concerns over immune rejection [71]. However, recent studies revealing the abnormal epigenetics, genomic stability, and immunogenicity of iPSCs have raised safety concerns over iPSC-based therapy [73, 74].

## 5. Therapeutic Applications of hESCs

As hESCs hold a lot of promises for the patients who are suffering from degenerative diseases, various attempts have been made to explore the therapeutic potentials in humans. The main objective of stem cell-based therapy is to restore or repair the lost or damaged body cells or tissues. To make hESCs suitable for clinical applications, the derived stem cells must be manufactured as per United States Food Drug Administration (USFDA), Current Good Manufacturing Practices (cGMP), and Guidelines for the Clinical Transplantation of Stem Cells, respectively [75]. The chemicals, reagents, cells, and machines and instrumentations used in the stem cell culture should undergo safety and health checks, and all manufacturing processes must be monitored and documented as per cGMP guidelines. If we analyze how many currently used hESC lines comply with the cGMP guidelines, you will find that many of the hESC lines will fail to meet the cGMP guidelines, because many hESCs are exposed to immunogenic or pathogenic animal components during their isolation and propagation stages. Another reason for failing to meet the cGMP guidelines is that most of hESC culture works were conducted in a university's laboratories, wherein many of these research laboratories do not comply with the cGMP guidelines. Till today, only a few researchers could be able to produce hESC lines as per the cGMP guidelines [14, 76, 77].

Considering potential commercial benefits of hESCs, few biotechnology companies were also involved in funding the stem cell research with a sole aim of commercializing stem cell products. These companies have started manufacturing hESCs under cGMP conditions and started testing stem cells under clinical setting. In 2009, Geron Corporation (California-based biotechnology company) applied to FDA to start its first clinical trial using cells derived from hESCs. The clinical trial was started in October 2010, where 3 patients who were suffering from spinal injury were injected with 1.5 million oligodendrocyte precursor cells derived from hESCs [78]. The trial was unexpectedly discontinued and we do not know the reason, probably because preliminary results of the trial showed that the cells derived from hESCs did not result in any noticeable improvement in spinal injury. In addition, the FDA also approved another trial for the use of hESCs in macular degeneration disease [79–85]. Another company, Advanced Cell Technology located in Marlborough, Massachusetts, started clinical trials using hESCs. The cells were injected in the patients who were suffering from Stargardt's muscular dystrophy and from age-related dry macular degeneration. The retinal pigment epithelial (RPE) cells derived from hESCs were used [83]. In the study, RPE cells were administered in the patients, and after 4 months of posttransplantation, it was found that patients showed minor improvements in visual function without any indication of immune rejection or any sign of teratoma formation [83]. Stem cells were also tested in patients with type I diabetes, where pancreatic precursor cells were administered to the patients [86].

## 6. Summary and Conclusion

Human embryonic stem cells have great therapeutic potentials for the treatment of various diseases such as cancer, Parkinson's disease, Alzheimer's disease, and diabetes. Both in vitro and in vivo studies suggest that there is still hope that future embryonic stem cells will provide cures for various diseases. But the success of stem cell-based therapy depends on the availability of mature and functional cells. To obtain mature and functional cells, it would be better if stem cells are grown under three-dimensional (3D) culture condition. Most of the hESC lines are obtained through two-dimensional (2D) culture conditions. There are a few limitations of using 2D culture, as hESCs which have grown in 2D condition do not represent human cells of the human body and most of the 2D cultured hESCs are reported to die immediately after cell transplantation; those cells that survived still fail to repair the body tissues. This issue can be handled, by culturing hESCs in 3D conditions, where cells can grow in three directions and chances of cell survivability will enhance after cell transplantation. Another important point to consider for successful stem cell-based therapy is to rigorously evaluate stem cell-derived cells in animal models before testing in humans. The cell-cell integration, cell-cell communication, cell migration, and cell functionality need to be evaluated thoroughly in animal models using both short-term and long-term trial approaches. The issue related to trauma formation and immunorejection must also be resolved by developing stem cell lines which do not cause immunorejection and do not form tumor after transplantation. This can be achieved by silencing the gene/molecular pathways which trigger tumor formation and immunorejection, respectively. Moreover, the cell-based therapy also demands many mature cells, and efforts should also be directed towards isolation of large quantity of stem cells and their precursors by bringing a new innovative approach and methodology. Finally, human embryonic stem cells still hold a great promise for the treatment of various degenerative diseases as well as diagnostic applications.

## Figures and Tables

**Table 1:** Advantages and disadvantages of inner cell mass (ICM) isolation from human embryos.

Techniques to obtain ICM from human embryos	Advantages	Disadvantages
Mechanical dissection	Mechanical isolation of the ICM proved to be an effective way to derive new hESC lines. The technique is fast and does not require xeno-components	Very laborious and time consuming
Laser dissection	Laser-assisted biopsy is also the most promising technique for xeno-free isolation of the ICM	Expensive
Immunosurgery procedure	High rate of ICM isolation	Immunosurgery procedure requires culture media containing guinea pig serum, which is not suitable for the generation of clinical-grade hESC lines
Microdissection	Easy method to isolate ICM	Poor success rate
Minimized trophoblast cell proliferation (MTP)	To derive hESCs from normal, abnormal, and frozen and thawed embryos	Only 50% success

**Table 2:** List of chemicals used to enhance culture of hESCs.

Name of chemicals	References
Matrigel	[48]
	[49]
Fibronectin	[51]
	[52]
Laminin and collagen type IV	[51]
	[52]
E-cadherin	[13]
E-cadherin/laminin 521	[13]
Synthetically designed bed surface	Melkoumian et al., 2010
Corning Synthemax Surface, a synthetic acrylate surface conjugated with vitronectin	Kawase et al., 2014
Spider silk proteins	Wu et al., 2014

**Table 3:** Multilineage differentiation capabilities of ESCs.

Name of different cells	References
Adrenal cells and keratinocytes	[58]
Insulin-producing cells	[59]
Neuronal cells	[60]
	[61]
Cardiac cells	[62]
Liver cells	[63]
Islet-like organoid	[64]
